# Habitat Quality and Geometry Affect Patch Occupancy of Two Orthopteran Species

**DOI:** 10.1371/journal.pone.0065850

**Published:** 2013-05-31

**Authors:** Gilberto Pasinelli, Kim Meichtry-Stier, Simon Birrer, Bruno Baur, Martin Duss

**Affiliations:** 1 Swiss Ornithological Institute, Sempach, Switzerland; 2 Department of Environmental Sciences, Conservation Biology, University of Basel, Basel, Switzerland; Ben-Gurion University of the Negev, Israel

## Abstract

Impacts of habitat loss and fragmentation on distribution and population size of many taxa are well established. In contrast, less is known about the role of within-patch habitat quality for the spatial dynamics of species, even though within-patch habitat quality may substantially influence the dynamics of population networks. We studied occurrence patterns of two Orthopteran species in relation to size, isolation and quality of habitat patches in an intensively managed agricultural landscape (16.65 km^2^) in the Swiss lowland. Occurrence of field crickets (*Gryllus campestris*) was positively related to patch size and negatively to the distance to the nearest occupied patch, two measures of patch geometry. Moreover, field crickets were more likely to occur in extensively managed meadows, meadows used at low intensity and meadows dominated by *Poa pratensis*, three measures of patch quality. Occurrence of the large gold grasshopper (*Chrysochraon dispar*) was negatively related to two measures of patch geometry, distance to the nearest occupied patch and perimeter index (ratio of perimeter length to patch area). Further, large gold grasshoppers were more likely to occupy patches close to water and patches with vegetation left uncut over winter, two measures of patch quality. Finally, examination of patch occupancy dynamics of field crickets revealed that patches colonized in 2009 and patches occupied in both 2005 and 2009 were larger, better connected and of other quality than patches remaining unoccupied and patches from which the species disappeared. The strong relationships between Orthopteran occurrence and aspects of patch geometry found in this study support the “area-and-isolation paradigm”. Additionally, our study reveals the importance of patch quality for occurrence patterns of both species, and for patch occupancy dynamics in the field cricket. An increased understanding of patch occupancy patterns may be gained if inference is based on variables related to both habitat geometry and quality.

## Introduction

Loss and fragmentation of habitats are among the main threats to biodiversity worldwide (e.g. [1]). Negative effects of these two processes have been shown for many taxonomic groups, including plants [2], insects [3], reptiles [4], birds and mammals [5]. Habitat loss results in a reduction of suitable habitat and has direct, negative effects on the population sizes of essentially all taxa. Habitat fragmentation breaks up suitable habitat and leads to a reduction of habitat patch size and an increase of patch isolation. The effects of habitat fragmentation appear to be more subtle than those of habitat loss and need not necessarily be negative [1]. In addition to habitat loss and fragmentation, habitat degradation is a third process affecting distribution patterns and spatial dynamics of species (e.g. [6]). Habitat degradation may arise from a relatively abrupt reduction of habitat quality over a short period or from a gradual decrease of habitat quality over time (see examples in [7]). All else remaining equal, habitat degradation will erode population sizes and therefore pose serious threats to the persistence of habitat specialists and to the maintenance of biodiversity. Although the importance of habitat quality in conservation is generally acknowledged and has been addressed to some extent in particular taxa (e.g. insects), far more studies have focused on habitat loss and habitat fragmentation than on the effects of habitat degradation [6]. Two recent reviews evaluated fragmented animal populations [8] and metapopulation dynamics [9] in relation to habitat area and isolation, but not to habitat degradation or quality. This is indicative of the relative paucity of studies on the combined effects of the three processes on plant and animal populations and may explain why fragmentation effects still are poorly understood and often controversial [7].

The aims of this study were to examine whether and how patch size, patch isolation and proxies of patch habitat quality affect the occurrence of two Orthopteran species, the field cricket (*Gryllus campestris*) and the large gold grasshopper (*Chrysochraon dispar*), in an intensively managed agricultural landscape in the Swiss lowland. Orthopterans are ideal organisms to assess occurrence patterns in relation to environmental conditions because these species are comparatively easy to monitor and respond quickly to environmental changes owing to their short generation times (one year). The following predictions relating to habitat geometry (i.e. patch size and isolation) were evaluated. First, based on metapopulation theory [10] and on previous studies on related species (e.g. bush cricket *Metrioptera bicolor*, [11],wart-biter *Decticus verrucivorus*, [12]), we predicted that occurrence of both Orthopteran species was positively related to patch size. Second, patch occupancy has been shown to be affected by patch isolation (e.g. [12], [13]). Increased distances among remnant habitat patches can impede dispersal of individuals, resulting in reduced migration rates among patches particularly for relatively immobile species. An insufficient exchange among occupied patches is considered a key mechanism underlying the negative relationship between patch occupancy and isolation (e.g. [7], [14]). We therefore expected occurrence of each species in a patch to be negatively related to the nearest distance to the closest occupied patch and positively related to an area-informed isolation metric, the proximity index [15], [16]. Apart from possible influences of habitat geometry, we anticipated relationships between species occurrence in a patch and proxies of habitat quality, which were selected based on known habitat preferences of each species [17], [18]. We hypothesized to find relationships between species occurrence and selected types of ecological compensation areas (ECA, see Method section for explanations), vegetation types and the presence of unmown vegetation in a patch. More specifically, we expected positive relationships between field cricket occurrence and ECA types such as extensively managed meadow or meadow used at low intensity (see Table 1) and vegetation types such as meadows characterized by *Poa* sp. or *Arrhenatherum* sp. (Table 2). Such ECAs and vegetation types, respectively, are characterized by low and sparse vegetation layers preferred by crickets [18]. For the large gold grasshopper, we expected positive relationships between species occurrence and patches with meadows with pithy plants (Table 2) and with the presence of unmown vegetation. Both factors are considered important for the presence and persistence of large gold grasshoppers, as females lay their eggs in stems of pithy plants, which persist in unmown vegetation to next spring [17]. Furthermore, we expected occurrence of the large gold grasshopper to be negatively related to distance of a patch to water, because this species appears to be associated with humid habitats [19], [20], which provide the plant species required for oviposition (see above). In contrast, no relationship was predicted between the occurrence of the field cricket, a species avoiding wet habitats [20], and distance of a patch to water. Finally, we expected positive relationships between the occurrence of both species and the age of the patch, as patch age has been shown to positively affect occurrence patterns in wood crickets (*Nemobius sylvestris*) [21]. Alternatively, the suitability of a patch may increase over the first years and then decrease again with ongoing succession. Therefore, we also examined whether species occurrence showed a quadratic relationship with patch age.

**Table 1:** Characterization of ECA types^a^ used in this study.

ECA type	Definition	n^b^	Size^c^
Extensively managed meadow	Meadows not fertilized and not mown before 15 June; usually 2 to max. 3 cuts per year; duration of cultivation = min. 6 years	232	0.34 (0.002 – 2.6)
Meadow used at low intensity	Meadows slightly fertilized, mostly with manure (max. 15 kg N ha^−1^ year^−1^) and not mown before 15 June; duration of cultivation = min. 6 years	38	0.30 (0.06 – 1.6)
Hedgerow with herbaceous strip	Herbaceous strip along hedgerows not fertilized and cut once per year (in sections); duration of cultivation = min. 2 years	37	0.16 (0.03 – 0.65)
Litter meadow	Meadows not fertilized and not mown before 1^th^ September; max 1 cut per year and min. 1 per three years; duration of cultivation = min. 6 years	35	0.25 (0.02 – 0.98)
others	Patch types not matching the above categories. Includes e.g. wildflower strips and extensively used pastures.	10	0.37 (0.02 – 1.1)

a ECA  =  ecological compensation area; ^b^ n =  number of patches; ^c^ size  =  mean (min. – max.) area of patches in ha.

**Table 2:** Characterization of vegetation types used in this study following [48].

Vegetation type	Characterization	n ^b^	size ^c^
Lolium a	Meadow with *Lolium multiflorum* or *Alopecurus pratensis* as dominant plant species. Nutrient rich soil, dense vegetation.	75	0.37 (0.01 – 1.9)
Poa	Meadow with *Poa pratensis* as dominant plant species. Soil with lack of manganese causing sparsely, low growing vegetation.	43	0.39 (0.01 – 1.7)
Arrhenatherum	All other meadows, often dominated by *Arrhenatherum elatius*. Soils with low nutrient concentration, intermediately dense vegetation.	126	0.25 (0.002 – 2.6)
Phalaris	*Phalaris arundinacea* is the dominant plant species.	30	0.34 (0.01 – 1.2)
Carex	Min. 50% of the ground is covered by low growing *Carex spp*.	20	0.43 (0.04 – 1.0)
other	High and dense vegetation: wildflower strips, margins and large growing sedges.	58	0.22 (0.03 – 1.1)

a The category name “Lolium” was chosen, because there were 63 patches with *Lolium multiflorum* as the dominant plant species, but only 12 patches with *Alopecurus pratensis*; ^b^ n  =  number of patches; ^c^ size  =  mean (min. – max.) area of patches in ha.

We additionally investigated patch occupancy dynamics by field crickets in relation to size, isolation and proxies of habitat quality of patches. This was possible because field crickets had been monitored in both 2005 and 2009, thereby providing the opportunity to address patch occupancy and turnover (colonization/extinction). According to the “area-and-isolation paradigm” (e.g. [13]), we expected patches that became colonized between 2005 and 2009 to be larger and better connected in 2009 than patches that remained unoccupied. Both increased patch size and increased connectivity promote successful dispersal among patches (e.g. [22], [23], [24]). In contrast, patches from which the species disappeared were predicted to be smaller and less connected in 2009 than patches remaining occupied. Both small size and low connectivity of patches can increase extinction risk. Populations in small patches are more at risk to be wiped out by stochastic events than populations in large patches [25]. Low connectivity reduces the chances that immigrants reach local populations before or after they go extinct. Patches occupied in both 2005 and 2009 were expected to be larger and better connected in 2009 than patches never occupied. We further examined whether patch occupancy and turnover were related to proxies of habitat quality [26], [27], [28] in 2009, expecting i) colonized patches to offer higher-quality habitat compared to patches remaining unoccupied, ii) extinct patches to offer lower-quality habitat compared to patches remaining occupied and iii) patches occupied in both years to offer higher-quality habitat than never occupied patches. The importance of habitat quality for patch occupancy dynamics has repeatedly been shown in diverse taxa (e.g. [26], [27], [28], [29], [30]).

Agricultural land use has strongly intensified over the past decades in industrialized countries leading to substantial declines of farmland biodiversity (e.g. [31] and references therein). To halt and reverse the negative effects, agri-environment schemes (AES) were introduced in many countries from the early 1990s on. In Switzerland, an AES with several types of ecological compensation areas (ECAs) was developed (see Methods). Such ECAs have also been implemented in our study area. Detailed knowledge on the ecological needs of species is a prerequisite to implement ECAs such that the benefit to biodiversity is increased, which is one of the key aims of AES. Therefore, results of this study may help informing land managers, farmers, and policy makers about the characteristics of ECA for promoting the occurrence of field crickets and large gold grasshoppers.

## Methods

### Ethics Statement

All surveys were conducted with the permission of the land owners and were in line with federal and cantonal legislation.

### Study site

The study was carried out in Central Switzerland at the plain of Wauwil (1665 ha, 498–538 m a.s.l, 47°10’N, 8°01’E), an intensively cultivated agricultural landscape. Most of the study area is farmland consisting of cropland (mainly maize) and artificial grassland. It is an open countryside structured by several windbreak hedges and a few small woods (covering 2.6% of the total study area). The study area includes three small protected wetlands and a small lake, relicts of a former marsh. In ancient times, the plain hosted a lake of approx. 500 ha, which was drained in the 19^th^ century. Despite numerous land improvements (meliorations), the plain of Wauwil has been largely spared from urban developments (settlements, roads). Since 1993, ecological compensation areas have been implemented in the plain of Wauwil as a part of an agri-environment scheme (see below), resulting in increased habitat connectivity between 2002 and 2009 (Table 3).

**Table 3:** Number of patches in relation to the distance among them in 2002 and 2009.

Distance	0–5 m		5–10 m		10–20 m		20–50 m		>50 m		Total	
Year	n	%	n	%	n	%	n	%	n	%	n	%
2002	117	54.9	33	15.5	11	5.2	12	5.6	40	18.8	213	100.0
2009	293	83.2	11	3.1	3	0.9	13	3.7	32	9.1	352	100.0

### Ecological compensation areas (ECAs)

In 1993, the Swiss government introduced an agri-environment scheme (AES) to promote biodiversity in agricultural landscapes. As a part of the AES, farmers receive subsidies for the implementation and maintenance of ecological compensation areas (ECA). To receive subsidies, farmers are required to manage at least 7% of the farmland as one or multiple types of ECA. Information on the amount and types of ECAs implemented in our study area was provided by the government of the canton Lucerne. Some types of ECAs, as for example fruit trees or dry stone walls, are not relevant for the species examined and rarely occurred in the study area, so that they were excluded from this study. Instead, we focused on the ECA types described in Table 1, which were considered potentially suitable habitats for the study species. Because some types had very small sample sizes (< 5), we pooled them into a single category named “others”. ECAs considered in this study are the only potentially suitable habitats for the species investigated in our study area, which largely consists of an intensively cultivated agricultural matrix (see Study site) hostile for field crickets and large gold grasshoppers.

An ECA is defined by the type of management applied, as detailed in Table 1. The size of an ECA thus reflects the area of potential habitat. We refer to these ECAs as patches throughout the text. Correspondingly, patch size (ha) was equal to the size of an ECA. Total sample size was 352 patches.

### Study species

#### Field cricket

The field cricket favours warm and dry sites in grassland and ruderal areas [18], [20]. During warm weather in spring, males sing in front of their burrows to attract females. After copulation, eggs are laid into the soil and the larvae hatch within a few weeks [32]. Larvae hibernate in burrows. Adults die after reproduction in July [19], [20]. Both males and females are unable to fly which prevents them from dispersing over long distances [20]. Typically, less than 40 m are covered within an individual’s lifetime, even though some individuals may move beyond 100 m [33]. The field cricket is common in central and southern Europe, western Asia and North Africa [19] and currently not threatened in Switzerland [34]. However, intensive agricultural practices destroy the preferred habitats and habitat fragmentation is an additional threat [20].

#### Large gold grasshopper

The large gold grasshopper prefers humid sites covered by high and dense vegetation (primarily grasses), as for example wet meadows, bogs, areas along ditches and water bodies [19], [20]. However, individuals occasionally occur also at drier sites [20], [35]. Eggs are laid in pithy stems of, for example, rush (*Juncus* sp.), sedge (*Carex* sp.), cattail (*Typha* sp.), goldenrot (*Solidago* sp.) and rotten wood, and eggs persist there over winter. Both a lack of suitable oviposition sites and the removal of vegetation with pithy stems preclude recruitment of new individuals. As a consequence, the large gold grasshopper cannot persist in regularly mown areas [35], suggesting that the availability of suitable oviposition sites is a key factor for the persistence of the species [17]. Large gold grasshoppers are highly philopatric and generally spend all of their lifetime in an area with radius of approx. 40 m [36]. The large gold grasshopper is currently classified as potentially threatened in Switzerland [34], with habitat loss and fragmentation arising from intensive land use and urbanisation being the most serious threats [20].

### Orthopteran surveys

Patches were acoustically surveyed for both species on dry and warm days (>18°C) from 10 a.m. to 9 p.m. Under these environmental conditions, both species are highly vocal [37], [38]. The song of field crickets can be heard up to 50 m from the animal, while the song of large gold grasshoppers is comparatively less loud and less conspicuous, but still easy to perceive.

All 352 patches (i.e. ECAs, see below and Table 1) within the study area were checked twice in 2009 for presence/absence of each species. We considered a patch to be occupied, if at least on individual of the species of interest had been found during at least one of the two surveys. Each patch was inspected by slowly walking along one diagonal transect. Due to this procedure, time spent per unit transect length was approximately constant across patch sizes, even though total time spent per patch increased with patch size as a consequence of increasing transect length. Given their small sizes (Table 1), patches can be considered to be similarly well surveyed and their occupancy status (not density) adequately assessed. Singing males of the field cricket were mapped from 12 May to 4 June (first survey) and from 5 to 15 June (second survey), respectively. We mapped the occurrence of the large gold grasshopper from 8 July to 4 August (first survey) and from 5 August to 3 September (second survey), respectively. During the two last surveys, some meadows had already been cut, which is detrimental for many Orthopterans [39]. However, the cutting regime of ECAs requires that 10% of the vegetation is left uncut, serving as a refuge from where Orthopterans can spread over the patch again [40]. To allow for this, patches were mapped three weeks after mowing at the earliest.

### Habitat variables

All variables referring to habitat geometry were based on the landscape context in 2009.

### Variables related to habitat geometry

To assess relationships between species occurrence and factors describing habitat geometry, we focused on the distance of the focal patch to the nearest occupied patch (hereafter referred to as “distance to nearest occupied patch”), patch size, the perimeter index and the proximity index. Distance to nearest occupied patch was the nearest edge-to-edge distance (m) of the focal patch to the closest patch occupied by a conspecific. Perimeter index was calculated as perimeter (m) divided by patch area (m^2^). An increasing perimeter index means increasing perimeter (and thus edge habitat) at constant patch size. The proximity index quantifies the spatial context of a habitat patch in relation to its neighboring patches [15] and is considered biologically realistic because it reflects the number of potential sources of dispersers that are close to a patch, as a function of their sizes and distances [16]. The proximity index was calculated as the sum, over all patches whose edges were within 50 m of the focal patch, of each patch size divided by the square of its distance from the focal patch [41]. The proximity index was computed with the V-LATE 1.1 extension for ArcGIS 9.2. We used 50 m as the threshold distance because this distance corresponds to the average movement radius of the study species [33], [36]. Increasing values of the proximity index indicate decreasing isolation of the focal patch. Distance to nearest occupied patch and the proximity index were used to examine the influence of patch isolation on cricket and grasshopper occurrence.

### Variables related to habitat quality of a patch

Vegetation structure and plant species composition may affect the suitability of a patch as habitat for Orthopterans. To allow inference on the role of habitat quality for species occurrence, each patch was assigned to one of six vegetation types (Table 2, “vegetation type”) based on the presence and abundance of characteristic plant species. These indicator species reflect the predominating abiotic conditions in a patch [42].

Likewise, each patch was assigned to one of five ECA types (Table 1, “ECA type”) to examine possible differences in their suitability as habitats for the study species. Additionally, the suitability of an ECA type may change over time due to succession, and the chance of a patch being occupied by dispersing individuals may increase, the longer the patch exists. Therefore, we assessed the number of years a patch was managed as ECA (“age”). Patch age ranged from 1 to 17 years, with a mean (and median) of 7 years.

The presence of senescent vegetation from the previous year serving as oviposition sites is important for the persistence of the large gold grasshopper (see above). We therefore recorded whether unmown vegetation of the previous year was present or not in each patch (binary independent variable “unmown vegetation”). Finally, we measured distance to water as the nearest edge-to-edge distance (m) of the focal patch to the closest water body.

### Patch occupancy dynamics

The occurrence of field crickets in the plain of Wauwil had already been mapped in 2005 with the same method used in 2009 (R. Graf pers. comm.). In 2009, 352 patches were surveyed. Of these 352 patches, 128 patches had already been surveyed in 2005. These 128 patches surveyed in both 2005 and 2009 thus allowed an analysis of patch occupancy dynamics. Each of the 128 patches was assigned to one of four categories: 1) occupied in both 2005 and 2009 (hereafter “patches occupied in both years”), 2) occupied in 2005, but not in 2009 (“extinct patches”), 3) occupied in 2009, but not in 2005 (“colonized patches”) and 4) patches not occupied in 2005 and 2009 (“unoccupied patches”).

### Data analysis

Statistical analyses were done using R 2.12.0. Because of skewed distribution, the variables distance to water, distance to nearest occupied patch and proximity index were log-transformed and patch size was square-root-transformed. All numerical variables were standardized to mean  = 0, SD  = 1 prior to the analyses. Standardized patch age was squared (age^2^) to test for a quadratic effect.

We examined the relationships between occurrence (binary dependent variable: 1 =  occurrence, 0 =  no occurrence) of the field cricket and the large gold grasshopper, respectively, in 2009 and the habitat variables outlined above for each species separately using generalized linear models (GLM) with binomial error distribution and logit link function (package stats). Patterns of patch occupancy are nowadays commonly analysed with site-occupancy models [43]. Site-occupancy models allow estimation of occupancy under consideration of detection probability, which is typically below 1. Therefore, estimates of occupancy with site-occupancy models are more robust than those based on traditional approaches not considering detection probability such as the analyses used here. However, detection probability requires repeated valid surveys of sites for calculation. We did not use site-occupancy models because the first cricket survey turned out to have been carried out too early in the season, when cricket activity was low and most patches were hence “unoccupied”. Applying site-occupancy models in such circumstances results in low detection probability estimates which in turn inflate occupancy probability estimates (M. Kéry, pers. comm.). To be consistent, we did not apply site-occupancy models to the large gold grasshopper data either.

Spatial structure may cause non-independence of data. We therefore evaluated the presence of spatial correlations in Orthopteran occurrence among patches with a semi-variogram of the residuals [44]. Because no evidence for spatial correlations was found (data not shown), no spatial correlation structure was included in the model.

We fitted separate GLMs including only two habitat variables and their 2-way-interaction to assess the importance of interactions. All possible two-way-combinations of habitat variables were analysed. For both species, none of the interactions was significant. In a second step, a GLM was fitted including all main effects. Significance of the habitat variables and the interactions, respectively, was assessed with likelihood ratio tests.

Intercorrelations among continuous habitat variables were examined with Pearson’s product moment correlation. Because some of the habitat variables were substantially correlated (see Results), we re-run the GLMs in step 2 by including only one variable of the correlated variable pair along with all the other habitat variables to see whether collinearity between the variables affected the outcome of the analyses. After that, another GLM was run now including the variable of the correlated variable pair excluded from the GLM just mentioned.

To assess the overall model fit we plotted the predicted values against the observed ones together with a running mean of the observations. To assess whether the quadratic model appropriately described the relationship between occurrence and patch age, we plotted the standardized residuals against age.

Patch occupancy dynamics was investigated with a linear discriminant analysis (LDA) to find out which variables explained differences among the four patch categories (see Patch occupancy dynamics). Variables considered in the LDA were patch size, distance to nearest occupied patch, perimeter index, proximity index, distance to water, age of patch, unmown vegetation, vegetation type and ECA type. Due to the reduced data set, sample size for most vegetation types was small. Hence, vegetation types were combined, resulting in the four groups: 1) the pooled vegetation types Carex and Poa, *2)* vegetation type Arrhenatherum, 3) vegetation type Lolium and 4) the pooled vegetation types Phalaris and others. In 2005, the 128 patches consisted only of the ECA type extensively managed meadow (Table 1). In 2009, some of these patches were no longer managed as extensively managed meadows, and the factor ECA type was therefore a binary variable (extensively managed meadow, others).

## Results

### General changes in extent of and distance among patches

Between 2002 and 2009, the total area of patches (i.e. ECAs) increased from 110 ha to 126 ha (i.e. from 6.6% to 7.6% of the study area), and the number of patches increased from 213 to 352 (Table 3). Distances among patches decreased, as 83.2% of all patches were within 0–5 m in 2009 as opposed to 54.9% in 2002 (Table 3).

### Correlations among habitat variables

Patch size and perimeter index were strongly correlated (r = –0.80, Table S1). In the large gold grasshopper, the correlation coefficient between distance to water and distance to nearest occupied patch was 0.55. Correlations among the other continuous habitat variables were weak (Table S1). Furthermore, boxplots did not reveal obvious associations between measures of patch geometry and patch quality (data not shown).

### Relationships between species occurrence in 2009 and patch characteristics

#### Field cricket

**Figure 1:**
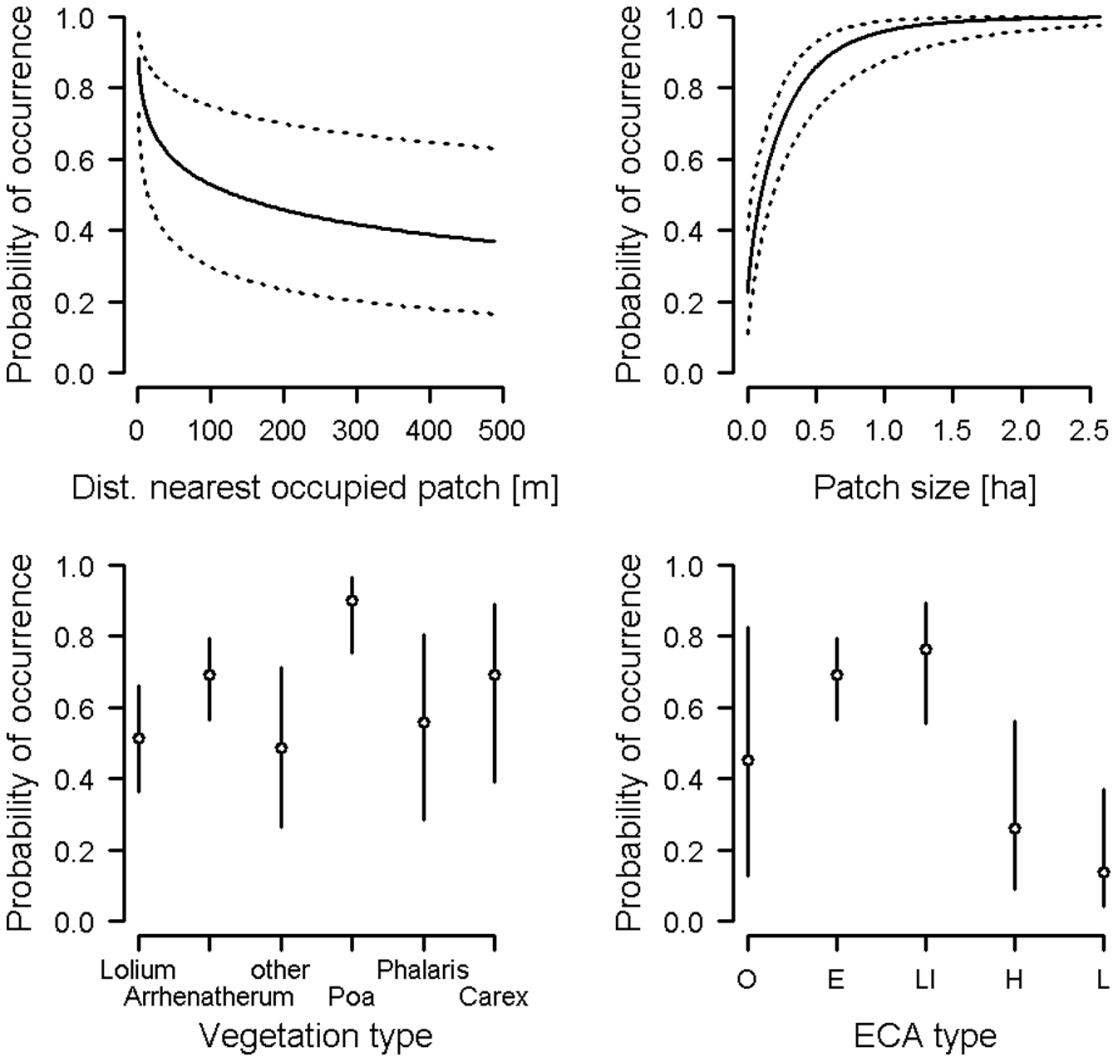
Patch occupancy in the field cricket. Probability of occurrence of the field cricket in a patch in relation to distance to the nearest occupied patch, patch size, vegetation type and ECA type (E  =  extensively managed meadow, LI  =  meadow used at low intensity, H  =  hedgerow with herbaceous strip, L  =  litter meadow, O  =  others). Plots show predicted values (solid lines and open circles) and 95% CI (dashed lines and vertical bars) from the model. N_occupied_  = 173, n_unoccupied_  = 179.

Occurrence of field crickets in a patch was significantly associated with four of the 10 habitat variables (Table 4). Occurrence was positively related to patch size and negatively related to distance to the nearest occupied patch (Table 4, Fig. 1). Moreover, field cricket occurrence was significantly related to ECA type and vegetation type (Table 4). ECA type "extensively managed meadow" was more likely to be occupied than the types “hedgerow with herbaceous strip” (*p*<0.03, Tukey post-hoc test; p value adjusted for multiple comparisons) and “litter meadow” (*p*<0.001). Likewise, ECA type "meadow used at low intensity" was more likely to be occupied than “hedgerow with herbaceous strip” (*p*<0.032) and “litter meadow” (*p*<0.001). Meadows of the vegetation type “Poa” were more likely to be occupied than vegetation types “Lolium” (*p*<0.005), “other” (*p*<0.007) and, as a tendency, “Phalaris” (*p* = 0.086) (Fig. 1). None of the other variables was significantly associated with the occurrence of the field cricket (Table 4).

**Table 4:** Relationships between field cricket occurrence and habitat variables.

Category	Subcategory	Estimate	SE	df	χ^2^	*p*
Intercept		–1.678	0.90	–	–	–
Patch size		1.174	0.23	1	31.2	0.001
Perimeter index		–0.128	0.20	1	0.46	0.497
Distance to nearest occupied patch		–0.853	0.16	1	30.9	0.001
Proximity index		0.077	0.14	1	0.29	0.588
ECA type				4	31.1	0.001
	extensively managed meadow	1.000	0.85			
	hedgerow with herbaceous strip	–0.844	1.02			
	meadow used at low intensity	1.364	0.96			
	litter meadow	–1.653	1.02			
	others	0	–			
Vegetation type				6	19.6	0.001
	Arrhenatherum	0.864	0.50			
	Carex	0.869	0.76			
	Lolium	0.109	0.54			
	Phalaris	0.296	0.68			
	Poa	2.253	0.65			
	other	0	–			
Distance to water		0.133	0.17	1	0.58	0.446
Unmown vegetation		0.620	0.35	1	3.13	0.077
Age		0.083	0.50	1	0.03	0.869
Age^2^		–0.094	0.48	1	0.04	0.846

Shown are parameter estimates and likelihood ratio tests from a GLM. N_occupied_  = 173, n_unoccupied_  = 179. Estimate of the variable unmown vegetation gives the difference between ”no unmown vegetation present” and "unmown vegetation present".

The separate analyses excluding from the GLM either of the two correlated variables patch size or perimeter index revealed that perimeter index became significant in the model without area (perimeter index: estimate  = –0.92, SE  = 0.21, likelihood ratio test χ^2^ = 27.7, *p* = 0.001), while all the other results did not change.

#### Large gold grasshopper

**Figure 2:**
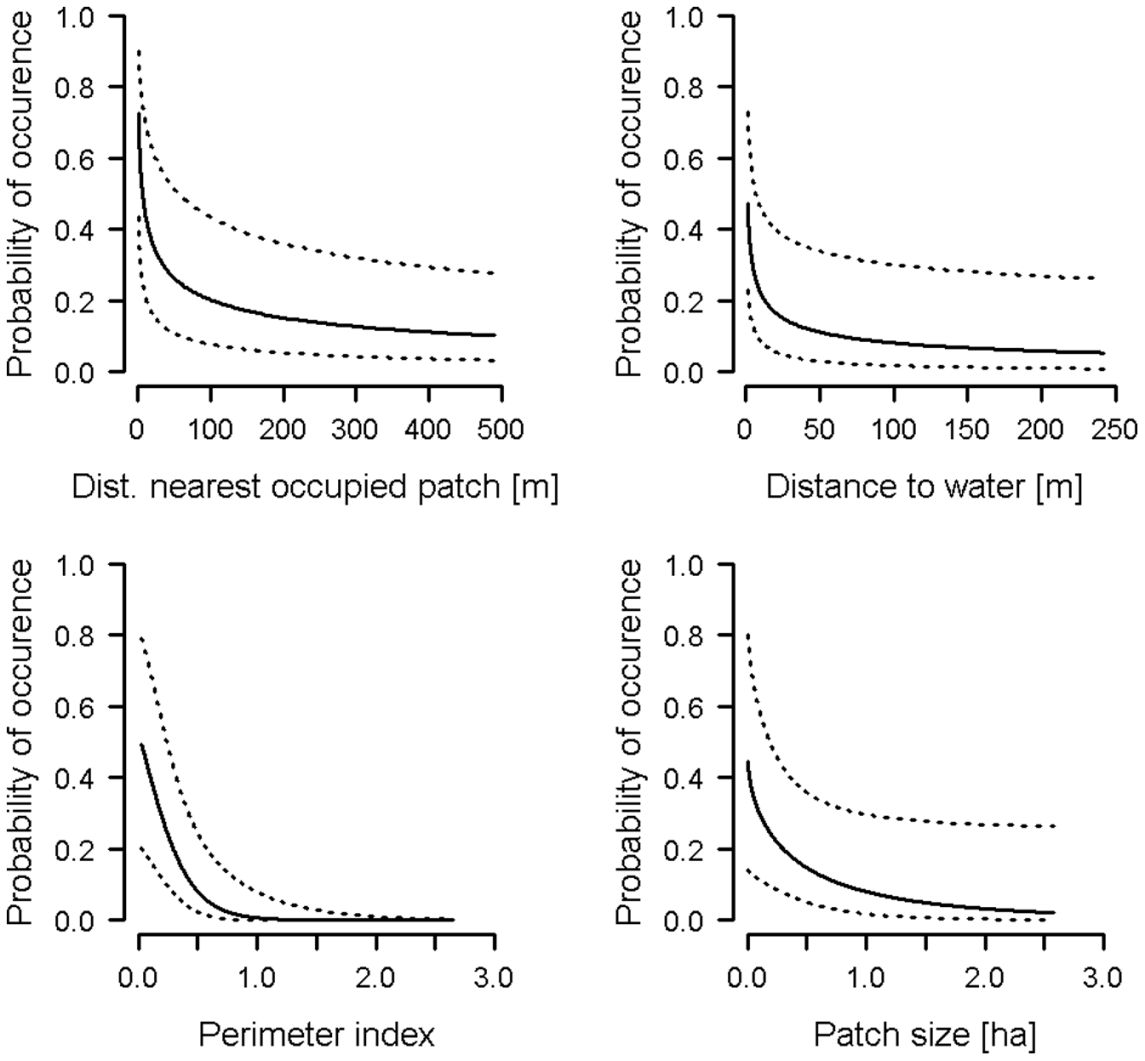
Patch occupancy in the large gold grasshopper. Probability of occurrence of the large gold grasshopper in a patch in relation to distance to the nearest occupied patch, distance to water, patch size and the perimeter index. Plots show predicted values (solid lines) and 95% CI (dashed lines) from the model. N_occupied_  = 71, n_unoccupied_  = 281.

Occurrence of the large gold grasshopper was significantly related to five of the 10 habitat variables (Table 5). Occurrence probability decreased with increasing values of the following variables: distance to the nearest occupied patch, distance to water, perimeter index and patch size (Fig. 2, Table 5). In addition, large gold grasshoppers were more likely found in patches with unmown vegetation than in patches without (mean probability of occurrence and associated 95% CI predicted by the model: with unmown vegetation: 0.265, 0.097–0.548; without unmown vegetation: 0.030, 0.008–0.108). All other habitat variables were not significantly related to the occurrence of the large gold grasshopper (Table 5).

**Table 5:** Relationships between large gold grasshopper occurrence and habitat variables.

Category	Subcategory	Estimate	SE	df	χ^2^	*p*
Intercept		–2.187	1.75	–	–	–
Patch size		–0.596	0.31	1	4.00	0.046
Perimeter index		–1.364	0.42	1	14.9	0.001
Distance to nearest occupied patch		–1.309	0.24	1	35.7	0.001
Proximity index		0.062	0.23	1	0.07	0.789
ECA type					6.66	0.155
	extensively managed meadow	0.784	1.73			
	hedgerow with herbaceous strip	–1.421	1.88			
	meadow used at low intensity	0.907	1.92			
	litter meadow	0.198	1.85			
	others	0	–			
Vegetation type				6	3.93	0.559
	Arrhenatherum	0.150	0.76			
	Carex	1.358	1.04			
	Lolium	–0.360	0.87			
	Phalaris	–0.261	0.85			
	Poa	–0.067	0.85			
	other	0	–			
Distance to water		–1.112	0.39	1	9.45	0.002
Unmown vegetation		–2.145	0.53	1	18.3	0.001
Age		0.911	0.96	1	0.93	0.336
Age^2^		–0.803	0.88	1	0.85	0.358

Shown are parameter estimates and likelihood ratio tests from a GLM. N_occupied_  = 71, n_unoccupied_  = 281. Estimate of the variable unmown vegetation gives the difference between ”no unmown vegetation present” and "unmown vegetation present".

When we excluded from the GLM either patch size or perimeter index (i.e. one of the two highly correlated variables, Table S1), the significantly negative effect of perimeter index remained (estimate ± SE  = –0.78±0.25, *p*<0.001, χ^2^ = 12.7), when excluding patch size. However, the effect of patch size became non-significant (0.25±0.19, χ^2^ = 1.8, *p*>0.186), when excluding perimeter index. In both GLMs, the relationships between the occurrence of the large gold grasshopper and the other habitat variables did not change when excluding one of the two variables mentioned above.

When excluding either distance to water or distance to nearest occupied patch (cf. Table S1), both variables remained significant as in the full model (distance to water: estimate ± SE  = –1.8±0.32, χ^2^ = 45.5, *p*<0.001; distance to nearest occupied patch: –1.6±0.23, χ^2^ = 71.7, *p*<0.001). However, patch size no longer was significant when distance to water was excluded from the GLM. Excluding distance to nearest occupied patch did not affect results for the other variables in the analysis.

### Patch occupancy dynamics

Of the 128 patches surveyed in both years, the field cricket colonized 50 (39.1%) patches and went extinct in 11 (8.6%) between 2005 and 2009. 25 (19.5%) patches were occupied in both years, while 42 (32.8%) patches remained unoccupied. The proportion of patches occupied significantly increased between 2005 and 2009 from 28.1% (36 occupied out of 128) to 58.6% (75 of 128, 2-sample test for equality of proportions with continuity correction χ^2^ = 22.97, df  = 1, *p*<0.001).

**Figure 3:**
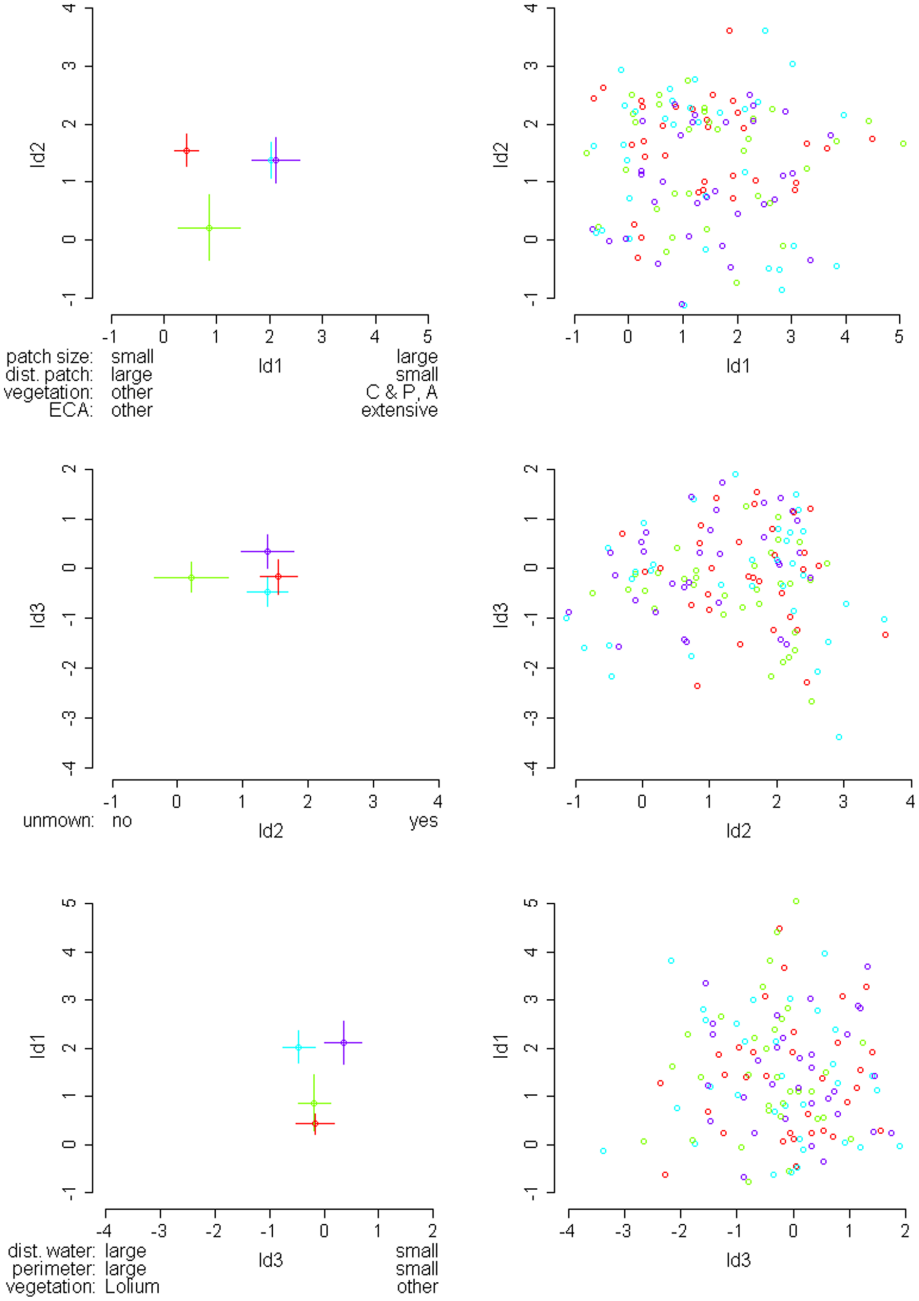
Differentiation of patch types based on three linear discriminant functions. Plots on the left show group means ±95% CI, plots on the right the discriminant function scores. For each linear discriminant function ( =  ld), the most influential variables according to Table 6 are indicated along with their specifications. Symbols: o  =  unoccupied patches, o  =  colonized patches, o  =  extinct patches, o  =  patches occupied in both years. Abbreviations: “dist. patch”  =  distance to nearest patch, “vegetation”  =  vegetation type (with C  =  Carex, P  =  Poa, A  =  Arrhenatherum), “ECA”  =  ECA type (extensive meadow), “unmown”  =  unmown vegetation, “dist. water”  =  distance to water, “perimeter”  =  perimeter index.

LDA revealed three discriminant functions which were used with the discriminant function coefficients of the nine variables (Table 6) and the group means with associated 95% CI (Fig. 3) to examine differences among the four patch categories. On the one hand, differentiation of patches occupied in both years, extinct patches, colonized patches and unoccupied patches was poor, when plotting discriminant scores of each patch based on the linear discriminant functions: the plots did not reveal any obvious separation of the four groups (Fig. 3, panels on the right). On the other hand, inspection of group means (and 95% CI) suggested a different interpretation. The first discriminant function, explaining 73.7% of the variance, separated patches occupied in both years and colonized patches from extinct and unoccupied patches, but did not distinguish patches occupied in both years from colonized patches and extinct from unoccupied patches (Fig. 3). The variables patch size, distance to nearest occupied patch, ECA type and vegetation type dominated the first discriminant function (Table 6). That is, patches occupied in both years and colonized patches were larger and closer to another occupied patch than extinct and unoccupied patches. Further, patches occupied in both years and colonized patches more often consisted of both the ECA type "extensively managed meadow" and the vegetation types "Carex and Poa pooled" and “Arrhenatherum*”* than extinct and unoccupied patches (Table 6, Fig. 3).

**Table 6:** Results of a linear discriminant analysis with four patch types (unoccupied, extinct, colonized, occupied in both years) as response variable and nine habitat variables as predictors.

Category	Subcategory	ldf1	ldf2	ldf3
Patch size		**0.694**	0.401	–0.147
Perimeter index		–0.157	0.171	–**0.435**
Distance to nearest occupied patch		–**0.437**	0.126	–0.034
Proximity index		0.260	–0.040	–0.028
ECA type	extensively managed meadow	**0.867**	0.006	–0.610
Vegetation type	Arrhenatherum	**0.751**	0.726	0.443
	Carex and Poa	**1.156**	–0.427	–0.184
	Lolium	0.446	0.965	–**1.551**
Distance to water		0.077	–0.024	–**0.820**
Unmown vegetation		0.159	**1.541**	1.066
Age		–0.174	0.090	0.257

For each predictor, the largest coefficient across the three linear discriminant functions (ldf) is printed in bold if >0.4 and used for interpretation of the respective ldf. N  = 128 patches. Coefficient for the variable unmown vegetation is for “unmown vegetation present”.

The second and third discriminant functions explained, respectively, 15.5% and 10.8% of the variance and thus contributed much less to the discrimination of the four patch categories than the first function. The second discriminant function separated extinct patches from the other three patch categories and was dominated by the variable unmown vegetation. Accordingly, extinct patches in 2009 more often lacked unmown vegetation than the other three patch categories (Table 6, Fig. 3). The third discriminant function separated, if anything, patches occupied in both years from colonized patches, since the 95% CI did not overlap the group means (Fig. 3). Colonized patches appeared to be located further from water, to have a larger perimeter index (higher edge-to-area ratio) and to consist more often of the vegetation type “*Lolium”* than patches occupied in both years (Table 6).

## Discussion

This study shows that the occurrence of two Orthopteran species is related to patch geometry and quality. The main factors influencing Orthopteran occurrence were patch size, distance to nearest occupied patch, distance to water, occurrence of unmown vegetation as well as types of ECA and predominant vegetation. Patch occupancy dynamics, only investigated for the field cricket, was driven by almost the same factors. Thus, size, connectivity and quality of patches were altogether of importance for the occurrence of both Orthopteran species and for patch occupancy dynamics of the field cricket.

### Patch geometry

The importance of patch size for species occurrence has been shown in many taxa (e.g. mammals: [30], insects: [45], spiders: [46], birds: [47]), even though the relationship between species occurrence and patch size may not always be strong [8]. In our study, the occurrence of the field cricket was positively related to patch size. The increase in occurrence was pronounced at small patch sizes, while patches of 1 ha or larger had a very high probability of being occupied (Fig. 1). In contrast, the relationship between patch size and occurrence was less clear in the large gold grasshopper. Patch size was only a significant predictor of this species’ occurrence, when perimeter index and distance to water (along with the other habitat variables) were included in the model. That is, the negative effect of patch size on grasshopper occurrence was only visible, when we accounted for the effects of perimeter index and distance to water by keeping the highly-correlated variables in the model. Perimeter index in turn was always significantly and negatively related to the occurrence of the large gold grasshopper, suggesting that this species more likely occupied patches with relatively little edge habitat (indicated by a low perimeter-to-area ratio).

Besides patch size, isolation of patches can negatively influence species occurrence across taxa (e.g. [21], [30]). In their comprehensive review, Prugh et al. [8] found stronger effects of distance to the nearest occupied source patch, a demographic isolation measure, on patch occupancy than of distance to nearest habitat patch of any size and distance to nearest large patch, two measures of landscape isolation. Our results mirror these general findings of Prugh et al. [8], in that a measure of demographic isolation (referring to the extent of exchange of individuals among patches), distance to nearest occupied patch, was a significant predictor of patch occupancy in both study species, while a measure related to landscape isolation, the proximity index, was not. As shown in Figures 1 and 2, the probability of occurrence of both species decreased rapidly with increasing distances to the nearest occupied patch. This suggests that for relative immobile species such as field crickets and large gold grasshoppers, high demographic connectivity on a small spatial scale is important for population expansion and probably persistence as well.

Prugh et al. [8] showed that the ability of patch area to predict occupancy is related to broadly defined species traits, such as taxonomic group (increasing effect from amphibians, reptiles, invertebrates, mammals to birds), diet (increasing effect from omnivores over herbivores to carnivores) and habit (stronger effect in species with arboreal than terrestrial lifestyles), but not to specialization. Moreover, none of these species traits affected the relationships between patch occupancy and isolation. Despite these emerging general patterns, the association of patch occupancy to patch geometry remains unclear for many species, which may be due to the omission of habitat quality in most studies assessing the influence of patch geometry on occupancy patterns.

### Habitat quality

Aside from factors relating to patch geometry, habitat quality of a patch may strongly affect species occurrence [6 and studies therein]. We found significant relationships between the occurrence of both Orthopteran species and proxies of patch habitat quality. The field cricket more often occurred in the two ECA types "extensively managed meadow" and "meadow used at low intensity" than in “hedgerow with herbaceous strip” and “litter meadow”. Moreover, field crickets were more often found in the vegetation type *“*Poa” than in the vegetation types “Lolium”, “other” and, as a tendency, “Phalaris” (see Fig. 1). ECA and vegetation types being more likely occupied are characterized by a relatively sparse and low-growing vegetation structure, which appears to provide favorable conditions for the field cricket, a species known to prefer warm and dry sites (e.g. [18]). Field crickets lay their eggs into self-made earth holes or on loose ground [20]. Thus, the preference for dry and warm sites may be linked to the requirements of eggs (and larvae), but further studies are needed to clarify the mechanisms underlying the above preference.

In contrast, the large gold grasshopper is usually found near ditches and in fields with wet ground [19]. In line with this, large gold grasshopper occurrence in our study was highest in patches close to water and steeply dropped with increasing distance from water. At least two non-exclusive hypotheses may explain the relationship between species occurrence and distance to water. First, patches close to water may provide suitable habitat, particularly for egg-laying (see next paragraph for a mechanistic explanation). Second, the relationship between species occurrence and distance to water may mirror the re-colonization patterns of the large gold grasshopper in the plain of Wauwil. Until mid-1990s, only few patches with unmown vegetation were available and these patches were located near water. We suspect that the large gold grasshopper persisted in these patches and dispersed from them when nearby habitat became available after the implementation of the AES in our study area.

Furthermore, large gold grasshopper occurrence was positively related to the presence of unmown vegetation. In our study area, patches near water are often covered with unmown vegetation, since they are hardly accessible and therefore difficult to mow. Unmown vegetation in humid patches (i.e. close to water) offers pithy stems of, for example, rush (*Juncus* sp.), sedge (*Carex* sp.) or cattail (*Typha* sp.), in which eggs are laid and persist over winter [17]. The species cannot persist in regularly mown areas [35] and so the survival of a local population in a patch might depend on whether such oviposition sites are left uncut over the winter.

### Patch occupancy dynamics

ECAs have been implemented in the study region since 1993 to reduce the negative effects of the intensive agricultural management on biodiversity. Both number and connectivity of the ECAs (i.e. patches) substantially increased from 2002 to 2009, resulting in a decrease of distances among patches (Table 3). The proportion of high-quality patches also increased from 5.5% to 18.6% (R. Graf unpublished). These developments seem to have favoured the field cricket, as this species was found in proportionately more patches in 2009 than in 2005. However, the separation of the four patch types (patches occupied in both years, extinct patches, colonized patches and unoccupied patches) was difficult, perhaps because ecological data used as predictors were available only from 2009. According to the LDA, colonised patches and patches occupied in both years were larger and better connected than patches going extinct and being never occupied. Similar to studies on bush crickets [11] and other organisms (e.g. [30]), our results suggest that habitat fragmentation affects the colonization/extinction dynamics of patches by the field cricket.

Besides factors related to patch geometry, patch quality turned out to be important for patch occupancy dynamics of the field cricket. Patches occupied in both years and colonized patches were characterized by extensively managed meadows (ECA type) and by meadows of the vegetation types “Carex and Poa” and “Arrhenatherum”. The associated vegetation structure ranged from low and sparsely growing (“Carex and Poa”) to intermediately dense and high (“Arrhenatherum”). Such ECA and vegetation types appear to be conducive to persisting and new local populations and mirror the findings from our analyses of patch occupancy. Thus, our study contributes to the increasing evidence that patch quality is important for patch occupancy dynamics in population networks of various taxa (e.g. [26], [27], [28], [30]).

We acknowledge that our study design may limit the generality of the above conclusions because no ecological data was available for the years 2005 to 2008 and no species occurrence data was available for 2006 to 2008. Colonization/extinction events might have occurred anytime within this period and need not necessarily be only related to the landscape context of 2009.

### Conclusions and conservation implications

Our study supports the “area-and-isolation paradigm” [13] in that we found strong relationships between Orthopteran occurrence and aspects of patch geometry, these being patch size (field cricket), perimeter index (large gold grasshopper) and distance to nearest occupied patch (both species). At the same time, our study reveals the importance of patch quality for occurrence patterns of both species. In terms of patch occupancy dynamics, addressed only in the field cricket, both patch geometry and patch quality turned out to be relevant. Collectively, these findings suggest that an increased understanding of patch occupancy patterns may be gained if inference is based on variables related to both habitat geometry and quality.

Our results can be used to inform farmers and conservation managers about where and how ECAs are best implemented and managed to promote the occurrence of the Orthopteran species studied. Field crickets and large gold grasshoppers need well-connected habitat patches to facilitate inter-patch movements, as both species generally disperse over only short distances [20], [33], [36]. Conservation strategies should thus aim at increasing the size of suitable habitat patches (>0.5 ha for field crickets) and habitat connectivity as well as maintaining or improving habitat quality. The latter includes patches with low and sparsely growing vegetation for the field cricket, whereas the presence of vegetation not mown over winter appears to be critical for the large gold grasshopper.

## Supporting Information

Table S1 **Pearson’s product moment correlation coefficients between continuous habitat variables.**
(DOC)
