# Supplementary material for: Habitat Quality and Geometry Affect Patch Occupancy of Two Orthopteran Species
Source: PLoS One. 2013 May 31;8(5):e65850. doi: 10.1371/journal.pone.0065850 (PMC3669274; doi:10.1371/journal.pone.0065850)
Supplement: Table S1 — Pearson’s product moment correlation coefficients between continuous habitat variables. (DOC) [file pone.0065850.s001.doc]

**Table S1.** Pearson’s product moment correlation coefficients between continuous habitat variables.

|  | **Patch size** | **Age** | **Distance to nearest occupied patch** | **Proximity index** | **Distance to water** | **Perimeter index** |
| --- | --- | --- | --- | --- | --- | --- |
| Patch size | - | -0.01 | -0.18 | 0.09 | -0.05 | -0.80 |
| Age | -0.01 | - | 0.03 | 0.02 | 0.01 | -0.01 |
| Distance to nearest occupied patch | -0.01 | 0.05 | - | -0.05 | 0.55 | 0.09 |
| Proximity index | 0.06 | 0.01 | 0.03 | - | -0.12 | -0.05 |
| Distance to water | -0.05 | 0.01 | 0.26 | -0.12 | - | -0.09 |
| Perimeter index | -0.80 | -0.01 | 0.03 | -0.01 | -0.09 | - |

Values below the diagonal refer to the field cricket, values above the diagonal to the large gold grasshopper. N=352.
